# Supplementary material for: Overexpression of PSAT1 Gene is a Favorable Prognostic Marker in Lower-Grade Gliomas and Predicts a Favorable Outcome in Patients with IDH1 Mutations and Chromosome 1p19q Codeletion
Source: Cancers (Basel). 2019 Dec 18;12(1):13. doi: 10.3390/cancers12010013 (PMC7016949; doi:10.3390/cancers12010013)
Supplement: Supplementary file 1 [file cancers-12-00013-s001.zip › cancers-642402-proof--supplementary/cancers-642402-proof-check-supplementary-.docx]

Supplementary Materials

Overexpression of *PSAT1* Gene is a Favorable Prognostic Marker in Lower Grade Gliomas and Predicts a Favorable Outcome in Patients with *IDH1* Mutations and Chromosome 1p19q codeletion

Shang-Pen Huang, Yung-Chieh Chan, Shang-Yu Huang, Yuan-Feng Lin


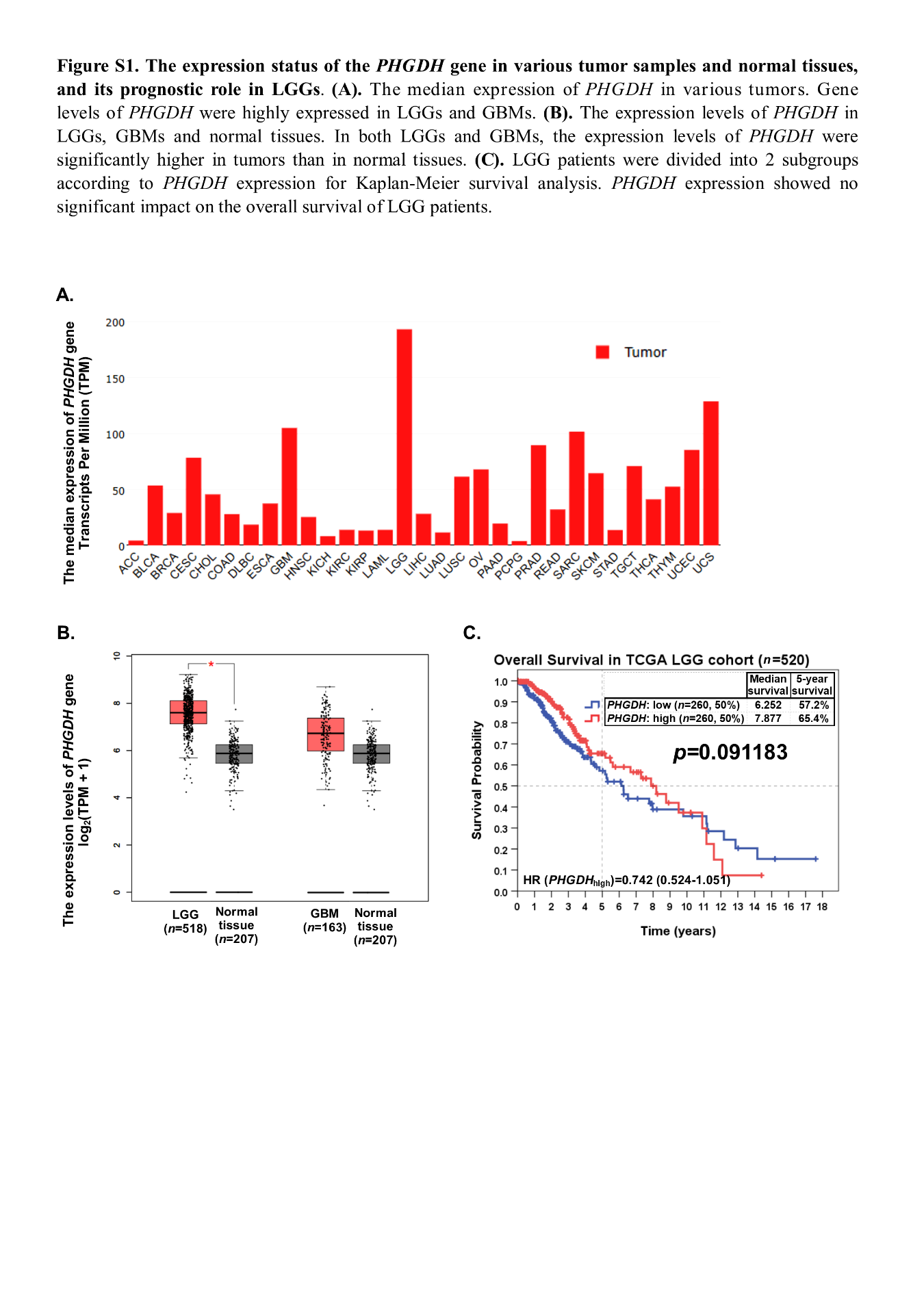


**Figure S1.** The expression status of the *PHGDH* gene in various tumor samples and normal tissues, and its prognostic role in LGGs. (**A**). The median expression of *PHGDH* in various tumors. Gene levels of *PHGDH* were highly expressed in LGGs and GBMs. (**B**). The expression levels of *PHGDH* in LGGs, GBMs and normal tissues. In both LGGs and GBMs, the expression levels of *PHGDH* were significantly higher in tumors than in normal tissues. (**C**). LGG patients were divided into 2 subgroups according to *PHGDH* expression for Kaplan-Meier survival analysis. *PHGDH* expression showed no significant impact on the overall survival of LGG patients.


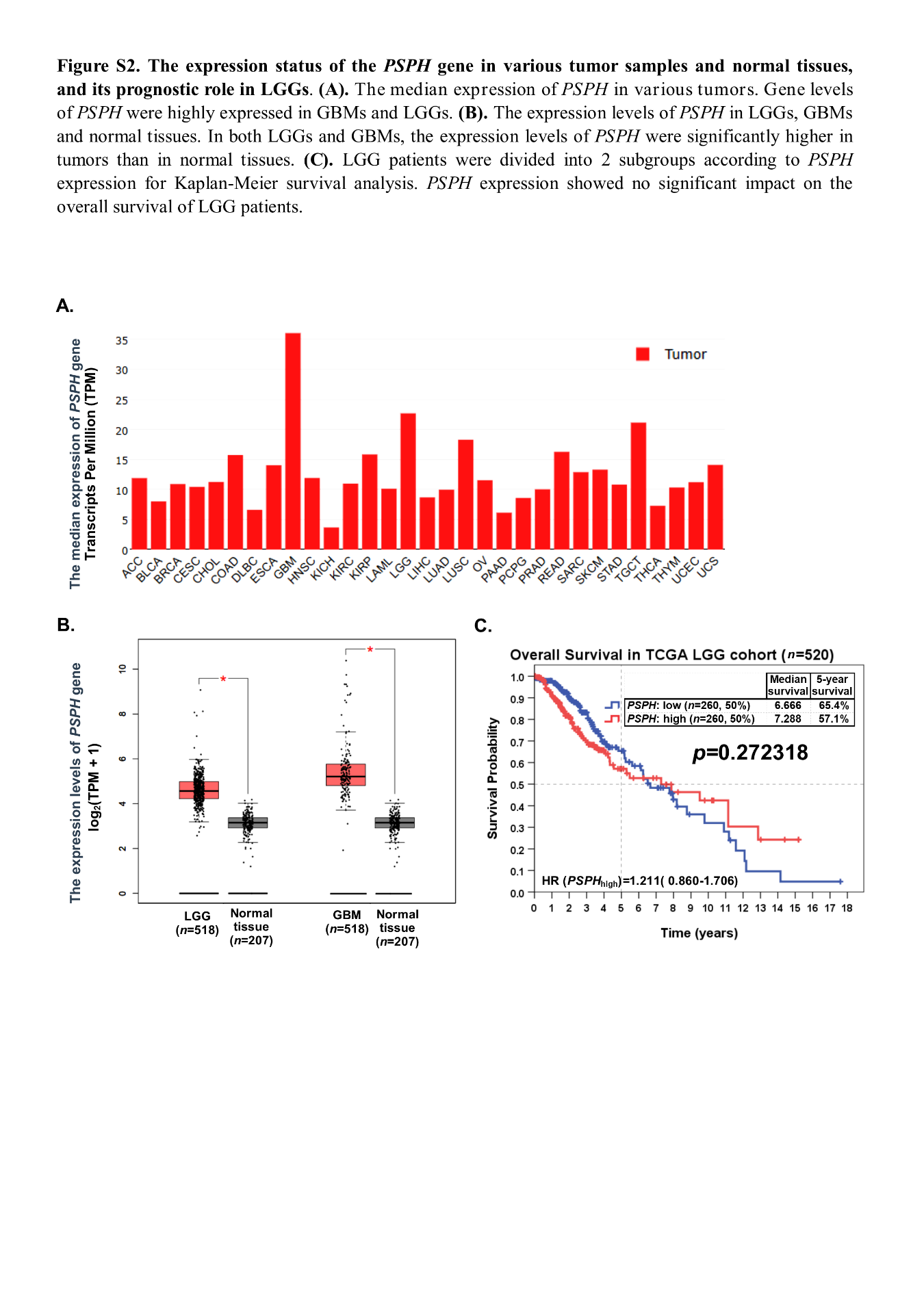


**Figure S2.** The expression status of the *PSPH* gene in various tumor samples and normal tissues, and its prognostic role in LGGs. (**A**). The median expression of *PSPH* in various tumors. Gene levels of *PSPH* were highly expressed in GBMs and LGGs. (**B**). The expression levels of *PSPH* in LGGs, GBMs and normal tissues. In both LGGs and GBMs, the expression levels of *PSPH* were significantly higher in tumors than in normal tissues. (**C**). LGG patients were divided into 2 subgroups according to *PSPH* expression for Kaplan-Meier survival analysis. *PSPH* expression showed no significant impact on the overall survival of LGG patients.


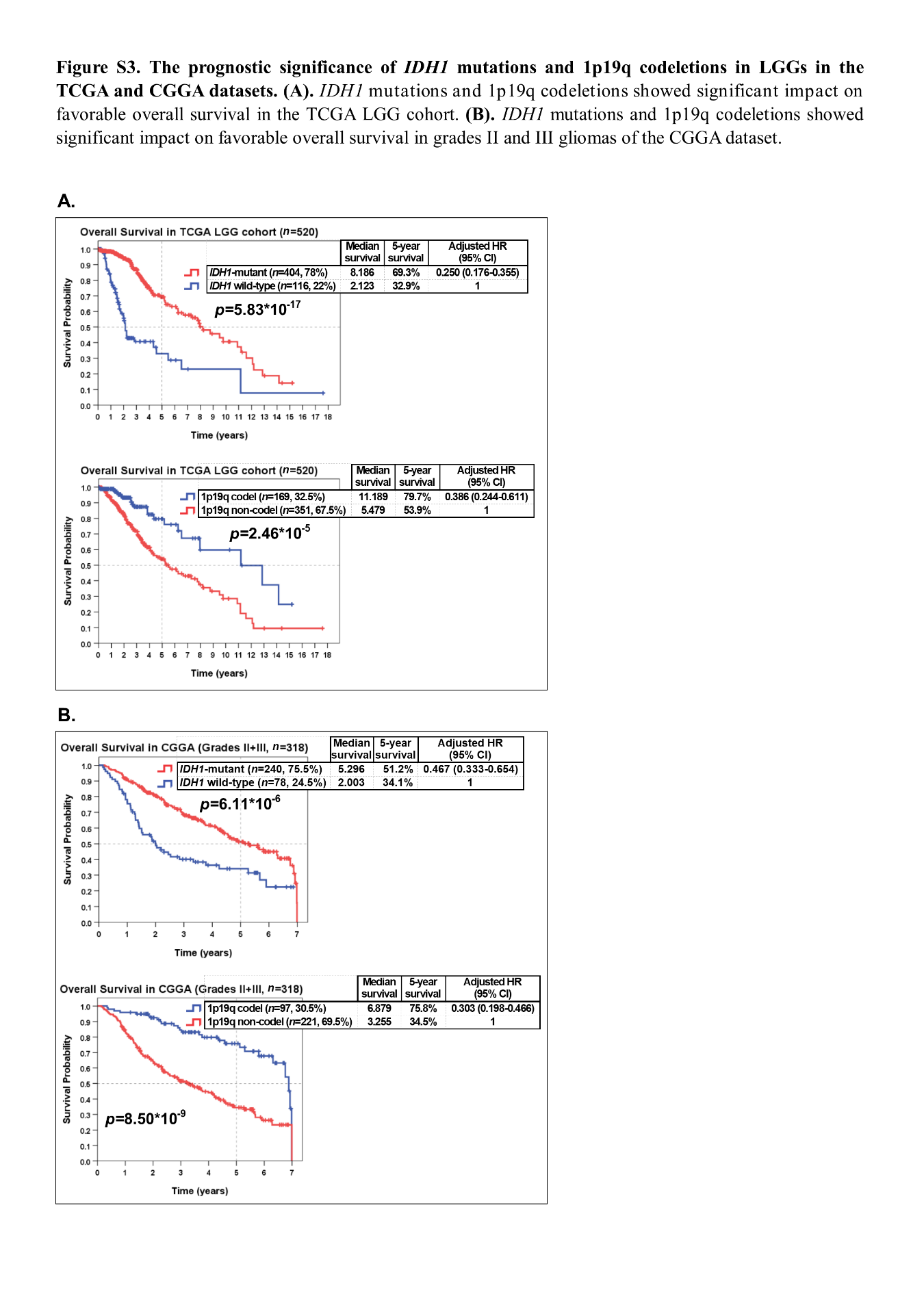


**Figure S3.** The prognostic significance of *IDH1* mutations and 1p19q codeletions in LGGs in the TCGA and CGGA datasets. (**A**). *IDH1* mutations and 1p19q codeletions showed significant impact on favorable overall survival in the TCGA LGG cohort. (**B**). *IDH1* mutations and 1p19q codeletions showed significant impact on favorable overall survival in grades II and III gliomas of the CGGA dataset.


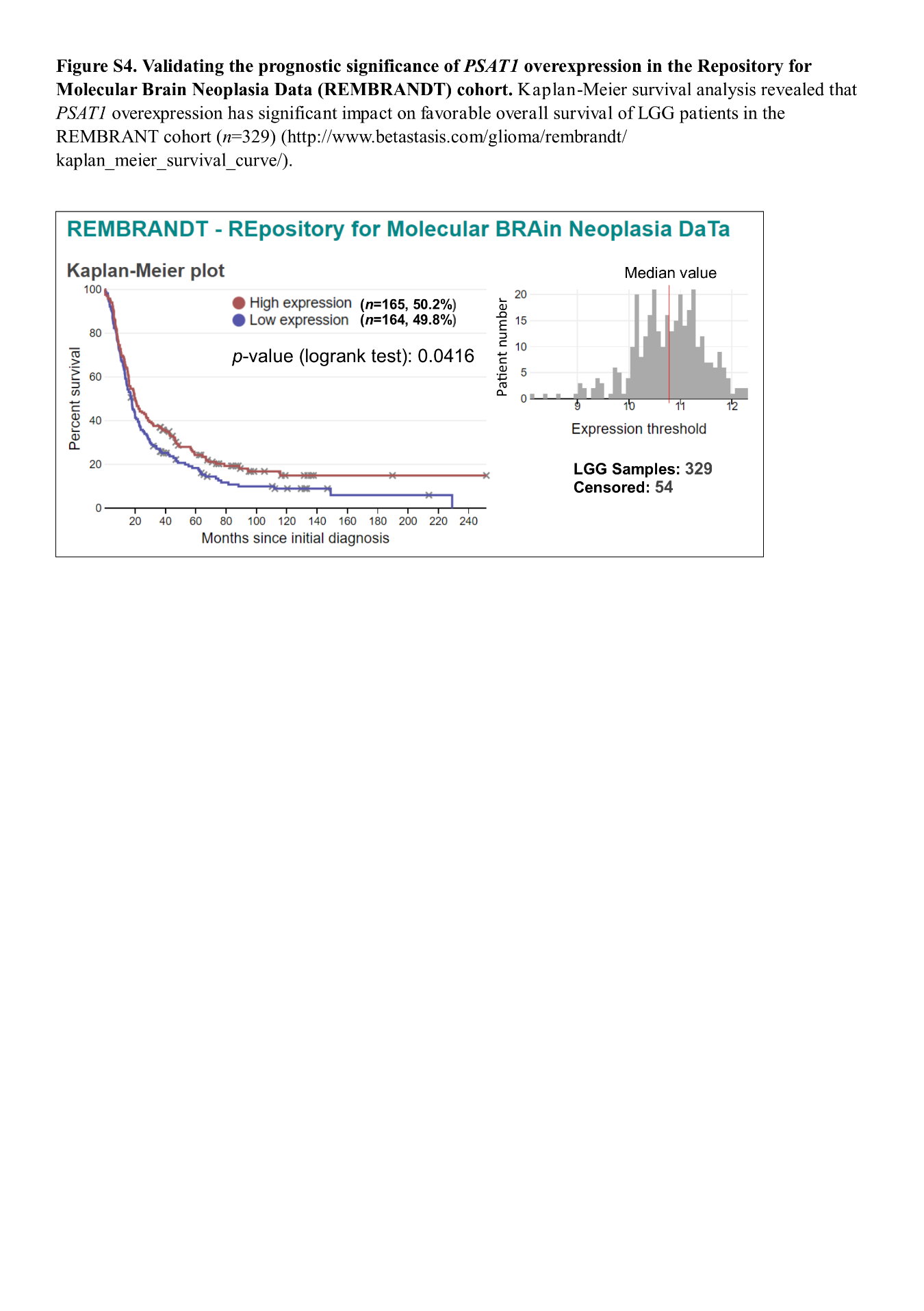


**Figure S4.** Validating the prognostic significance of *PSAT1* overexpression in the Repository for Molecular Brain Neoplasia Data (REMBRANDT) cohort. (A). Kaplan-Meier survival analysis revealed that *PSAT1* overexpression has significant impact on favorable overall survival of LGG patients in the REMBRANDT cohort (*n* = 329) (http://www.betastasis.com/glioma/rembrandt/kaplan_meier_survival_curve/).


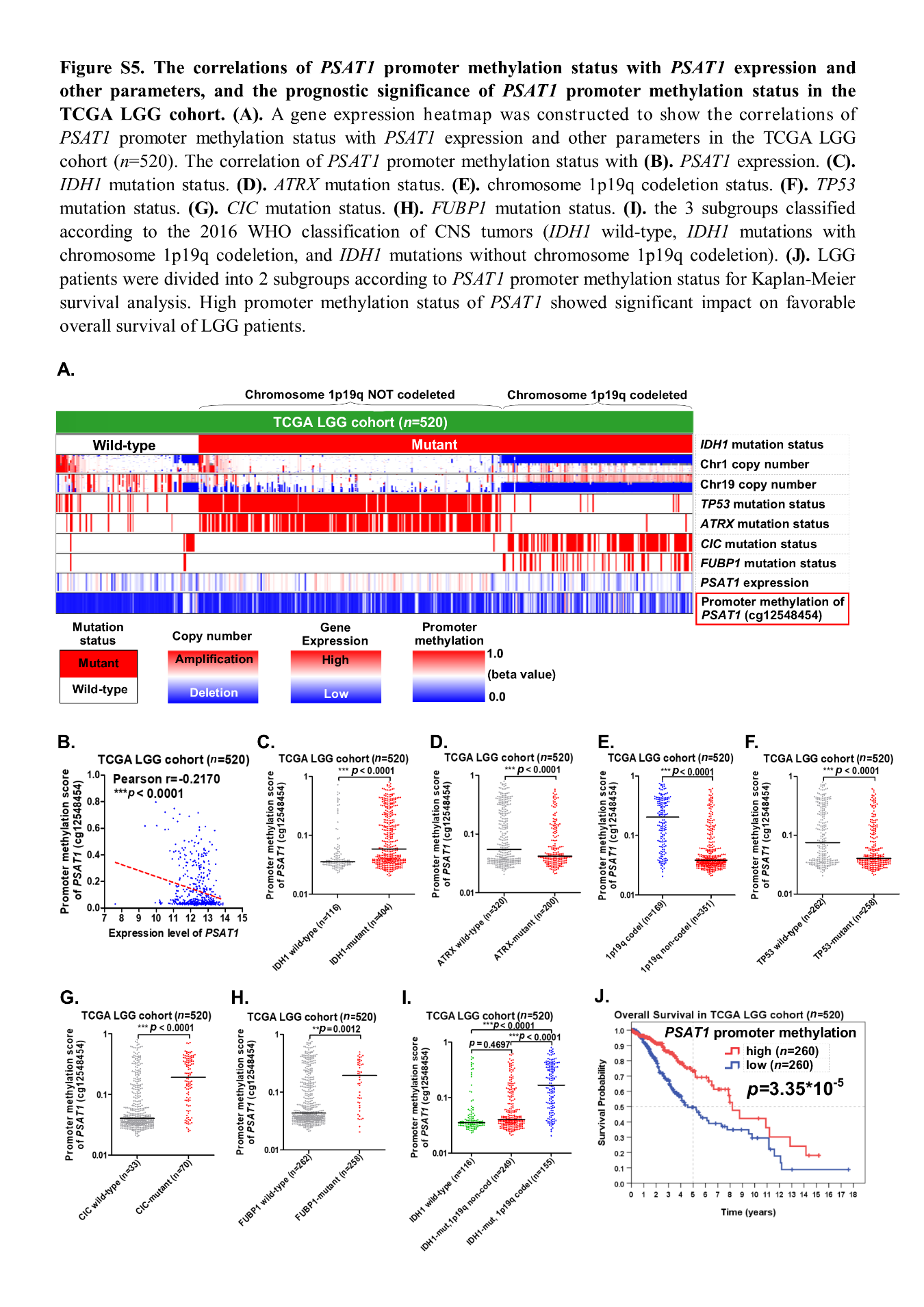


**Figure S5.** The correlations of *PSAT1* promoter methylation status with *PSAT1* expression and other parameters, and the prognostic significance of *PSAT1* promoter methylation status in the TCGA LGG cohort. (**A**). A gene expression heatmap was constructed to show the correlations of *PSAT1* promoter methylation status with *PSAT1* expression and other parameters in the TCGA LGG cohort (n=520). The correlation of *PSAT1* promoter methylation status with (**B**). *PSAT1* expression. (**C**). *IDH1* mutation status. (**D**). *ATRX* mutation status. (**E**). chromosome 1p19q codeletion status. (**F**). *TP53* mutation status. (**G**). *CIC* mutation status. (**H**). *FUBP1* mutation status. (**I**). the 3 subgroups classified according to the 2016 WHO classification of CNS tumors (*IDH1* wild-type, *IDH1* mutations with chromosome 1p19q codeletion, and *IDH1* mutations without chromosome 1p19q codeletion). (**J**). LGG patients were divided into 2 subgroups according to *PSAT1* promoter methylation status for Kaplan-Meier survival analysis. High promoter methylation status of *PSAT1* showed significant impact on favorable overall survival of LGG patients.

**Table S1–S2:** Please view at the excel file.

| 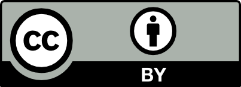 | © 2019 by the authors. Licensee MDPI, Basel, Switzerland. This article is an open access article distributed under the terms and conditions of the Creative Commons Attribution (CC BY) license (http://creativecommons.org/licenses/by/4.0/). |
| --- | --- |
